# Multi-isotope (*δ*^2^H, *δ*^13^C, *δ*^15^N) feather profiles and morphometrics inform patterns of migratory connectivity in three species of North American swallows

**DOI:** 10.1186/s40462-023-00412-2

**Published:** 2023-08-01

**Authors:** Keith A. Hobson, Kevin J. Kardynal

**Affiliations:** 1 Wildlife and Landscape Research Directorate, Environment and Climate Change Canada, Saskatoon, SK S7N 3H5 Canada; 2 Department of Biology, University of Western Ontario, London, ON N6A 5B7 Canada; 3 Department of Biology, University of Saskatchewan, Saskatoon, SK S7N 5E2 Canada

**Keywords:** Aerial insectivore, Isotopic niche space, Morphometrics, SIBER, Spatial clustering, Carbon-13, Nitrogen-15, Deuterium

## Abstract

Aerial insectivorous birds have suffered steep population declines in North America over the last 60 years. A lack of information on migratory connectivity between breeding and non-breeding grounds for these species limits our ability to interpret factors affecting their population-specific trends. We determined likely Latin American non-breeding regions of Bank (*Riparia riparia*), Barn (*Hirundo rustica*) and Cliff (*Petrochelidon pyrrhonota*) swallow from populations across their breeding ranges. We used predicted feather hydrogen (*δ*^2^H_f_) and carbon (*δ*^13^C_f_) isoscapes for winter-grown feathers to indicate areas of highest probability of moult origin and incorporated these results into a cluster analysis to determine likely broad non-breeding regions. We also assessed variation in wing length among populations to determine the potential for this metric to differentiate population moult origins. We then investigated patterns of multi-isotopic (*δ*^2^H_f_, *δ*^13^C_f_, *δ*^15^N_f_) and wing-length niche occupancy by quantifying niche size and overlap among populations under the assumption that broad niches were consistent with low within-species migratory connectivity and narrow and non-overlapping niches with higher connectivity. Multivariate assignment identified different non-breeding regions and potential clusters of moult origin generally corresponding to Central America and northern South America, eastern and south-central South America, and the western and southern part of that continent, with variation within and among populations and species. Separate niche space indicated different wintering habitat or areas used by species or populations whereas niche overlap indicated only potential spatial similarity. Wing length varied significantly among populations by species, being longer in the west and north for Bank and Cliff Swallow and longer in eastern Canadian Barn Swallow populations. Barn Swallow occupied consistently larger isotopic and wing length niche space than the other species. Comparisons among populations across species showed variable isotopic and wing-length niche overlap generally being greater within breeding regions and lower between western and eastern breeding populations supporting a general North American continental divide for all species with generally low migratory connectivity for all species. We present a novel approach to assessing connectivity using inexpensive and broad isotopic approaches that provides the basis for hypothesis testing using more spatially explicit expensive techniques.

**Supplementary Information:**

The online version contains supplementary material available at 10.1186/s40462-023-00412-2.

## Background

Migratory connectivity, the spatial and temporal linkages of migratory animals between their breeding and non-breeding areas, has received considerable attention and is clearly an important factor in shaping population dynamics of migratory species and populations [1–3]. At the mulitiple-population scale, migratory connectivity is considered strong when individuals from separate breeding populations spend the non-breeding period in the same spatially-defined region apart from other populations. In contrast, weak connectivity occurs when individuals from within discrete breeding or non-breeding populations have non-overlapping non-breeding or breeding regions, respectively, and mix with individuals from other populations [3, 4]. The degree of migratory connectivity can differentially impact individual survival or population-level trends due to exposure to various environmental variables such as land-use changes and climate change in different regions [2, 3, 5]. Understanding connectivity of migratory animals is therefore key for determining causes of population declines and implementing targeted conservation actions over the full annual cycle [6]. Unfortunately, migratory connectivity varies spatially and temporally and is difficult to quantify both conceptually and practically (but see [4, 6–8]).

In North America, swallows have experienced the steepest declines of all species groups over the last 60 years, a phenomenon shared generally by aerial insectivores [9–11]. Trends in swallow populations vary substantially across North America by species, and in general, several species have undergone declines in large parts of eastern North America and the northwestern part of the continent with less severe declines in west-central Canada and north-central United States [10, 12, 13]. Declining trends have resulted in two species, Bank (*Riparia riparia*) and Barn (*Hirundo rustica*) swallow being listed as Threatened on the Canadian Species at Risk Act (SARA) with populations of other swallow species also exhibiting declines across many regions. While Barn Swallow has recently been recommended for down listing by SARA due to stabilizing trends over the past several decades, their populations remain at significantly lower levels compared to 1960 [11]. Overall, causes of these population trends are unknown and likely differ by species and region but appear to be driven by multiple and potentially cumulative effects of habitat loss, pesticide impacts on prey and climatic variation, all of which vary continentally [9, 12–17].

Stable isotope measurements have been used effectively to delineate sources of nutrients, diets and trophic positions of a vast array of animal species and this field of research continues to expand at a rapid rate [18–22]. More recently, the analysis of stable isotopes in animal tissues has further allowed elucidation of spatial origins, especially for those tissues which are metabolically inert following formation [23–25]. The basis of this approach is that stable isotope patterns in foodwebs vary spatially and often can be modelled as predictable tissue-specific isoscapes [26–28]. Linking migratory animals spatially at various stages of their life cycle is critical to understanding their ecology and the stable isotope approach has several key advantages as an intrinsic marker. While this application is not new, we argue that isotopic niche space (19, 29–31) can provide insights into the strength of migratory connectivity between populations of organisms at various stages of their lifecycle. This idea follows on from the fact that isotopic niche breadth can be related to resource breadth and is expected to be higher with greater geographic or spatial range [32]. We reason that the isotopic niche holds the potential to quantify migratory connectivity of individuals and populations of species and that this aspect of isotopic analysis warrants further development and consideration as a method that can be applied to all migratory animals.

Using a multi-isotope (*δ*^2^H, *δ*^13^C, *δ*^15^N) approach based on winter-grown feathers, García-Pérez and Hobson [8] previously used a clustering approach to identify potential non-breeding regions of Barn Swallows in South America. That work suggested individuals from populations across North America had overall weak connectivity but did allude to a continental migratory divide later established with light-level geolocators [33]. Hobson and Kardynal [34] provided additional evidence of weak connectivity in eastern Canadian Barn Swallows by incorporating the use of stable sulfur isotope (*δ*^34^S) measurements and reporting on additional geolocator results. A single Bank Swallow and three Cliff Swallows (*Petrochelidon pyrrhonata*) fit with geolocators from a breeding population in New Brunswick in eastern Canada overwintered in northwestern South America and southeastern Brazil, respectively [35]. Origins estimated using feather stable isotopes from that same population for more individuals showed likely wintering areas largely concentrated in the Pampas ecoregion of Argentina and Brazil [35]. Studies of other swallow species in the western hemisphere show some continental structure in migration routes and non-breeding areas (e.g. Tree Swallow *Tachycineta bicolor*, [36]). However, although few, most previous studies suggest generally weak migratory connectivity between breeding and non-breeding areas across swallow species. Nonetheless, large gaps in our knowledge of most species exist due to a lack of broad-scale, range-wide studies.

Determining origins using isotopic assignment requires capturing and sampling an individual only once, is cost-effective relative to archival or transmitter tags, and depending on the nature of underlying isoscapes, can provide information similar to geolocators at broad scales [27, 37]. This approach to determining origins relies on the use of continental-scale gradients in naturally occurring stable isotopes in food webs [25, 38–40]. Deuterium (^2^H) in precipitation varies continentally according to well-described physical processes and these large-scale gradients of hydrogen isotopes in precipitation (*δ*^2^H_p_) and surface waters are transferred to metabolically inert animal tissues (e.g. feathers) via diet and drinking water. Stable carbon (*δ*^13^C) and nitrogen (*δ*^15^N) isotopes in food webs can also be used to refine isotopic assignment of individuals to origin because they show spatial patterning [41–43]. Foodweb *δ*^13^C is related to plant photosynthetic pathway, water-use efficiency, and climate [44]. This isotope has also been used to differentiate aquatic vs terrestrial foodwebs [18, 45, 46] but recent research indicates deuterium (i.e. *δ*^2^H) may be a more powerful tracer in this regard [47, 48]. Foodweb *δ*^15^N values are largely determined by plant nitrogen fixation mechanisms, fertilizer use and land-use practices (reviewed by [38, 49]). With probabilistic spatial models, combined use of multiple isotopes can be particularly insightful when spatial variation in a single isotope is not sufficient to assess origins across vast isotopic landscapes [8, 34, 50]. The use of stable isotopes to infer non-breeding origins is useful for several swallow species and other aerial insectivores because they molt during the non-breeding period [51, 52].

Multiple approaches using stable isotopes to estimate origins and migratory connectivity have been used in the Nearctic-Neotropical and Palearctic-Afrotropical systems [26]. Methods using multiple stable isotopes initially relied on clustering techniques, first using non-spatial and then spatially explicit clustering, which separated continents into isotopically similar regions (i.e. clusters; [8, 24, 53–56]). More recently, spatially-explicit approaches using multiple stable isotopes to assign individuals or populations to a single probability surface have been adopted [34]. A potentially promising and complementary method to this assignment approach is the use of Bayesian inference to assess variation in stable isotope profiles of individuals and populations to determine spatial segregation in regions where metabolically inert tissues are synthesized. Stable Isotopes using Bayesian Ellipses in R (SIBER) is a statistical tool that estimates heterogeneity within multi-isotope datasets incorporating variation in stable isotope profiles to determine isotopic niche space or volume [30]. Conceptually, the isotopic niche model can be applied to assess multi-isotope variation within and among populations of conspecifics and across species to assess migratory connectivity. The approach is based on the fact that populations occupying broad isotopic niche space, or multidimensional isotopic niche hypervolumes [29, 57], represent broad spatial locations or habitat types. In general, we suggest that for a given species or population, broad and overlapping isotopic niches will indicate low within-species or between-population migratory connectivity whereas narrow niches would indicate higher connectivity. Similarly, when comparing different species or populations, low niche overlap would indicate higher between-population or species connectivity. However, similar isotopic niches or high overlap do not necessarily indicate similar spatial origins of samples because identical isotopic profiles can occur at vastly different locations [58]. The power of the approach is very much in the direction of detecting low migratory connectivity within samples or low spatial overlap among samples. Nevertheless, the approach presents a potential quantitative means of defining migratory connectivity and could be powerful means of filtering population isotopic data to reveal spatial structure [59].

Morphometric traits of individual species often vary predictably across large spatial scales due to evolutionary requirements for life-history adaptations, which lends itself to potentially differentiating populations. Wing length and wing pointedness are particularly useful because they are associated with migration distance in many species including swallows [60–63]. Wing morphometrics have been used successfully in combination with probabilistic assignments using stable isotopes to determine provenance [64, 65] which requires prior knowledge of underlying spatial structure of wing morphology. Nonetheless, evidence of spatial variation in wing length can provide helpful insight into migratory behaviors and patterns.

The overall objective of this study was to apply a multi-isotope (*δ*^2^H_f_, *δ*^13^C_f_, δ^15^N_f_) approach to estimate non-breeding ground origins and migratory connectivity of several breeding populations of Bank, Barn and Cliff swallow in North America. In general, these species have broad breeding and wintering distributions and so have much scope for migratory segregation and overlap. All three species molt on the non-breeding grounds, which allows for approximation of their non-breeding origins in Latin America. Using two complementary spatial and non-spatial statistical approaches, we tested the hypotheses that: (1) conspecifics of discrete breeding populations wintered in different regions, and (2) different species from the same breeding region wintered in different regions. To determine likely regions of over-wintering origins of each population and species combination, we used a dual-isotope (*δ*^2^H, *δ*^13^C) probabilistic assignment-to-origin model constrained to the potential species-specific wintering areas. We used new stable isotope data collected from each species but also included published data analyzed by García-Pérez and Hobson [8] and Hobson and Kardynal [34] for Barn Swallows. Based on previous research [8, 33], we expected eastern populations of Barn Swallows to have isotopic profiles consistent with southern Brazil and northern Argentina whereas western populations were expected to have isotopic profiles consistent with Central America, the Caribbean and northern South America. Whether a similar east–west migratory divide occurs in the two other species is currently unknown due to limited migratory connectivity research on those species. We used spatial clustering of the assignment to origin probability surfaces to identify potential clusters of origin across populations for each species, separately. We also measured wing length of multiple breeding populations to assess variation among populations and to determine whether this variation was sufficient to differentiate population moult origins through inclusion in niche analyses. In general, we expected the more colonial-nesting species, Bank and Cliff Swallows, to show higher within-population (i.e. higher niche overlap) and lower between-population migratory connectivity (i.e. lower niche overlap) as shown in the Afro-Palearctic system for Sand Martin (*Riparia riparia*; [66]) compared to less colonial Barn Swallows and so occupy narrower isotopic niche space within populations [67–69].

## Methods

### Study areas and field methods

**Fig. 1:**
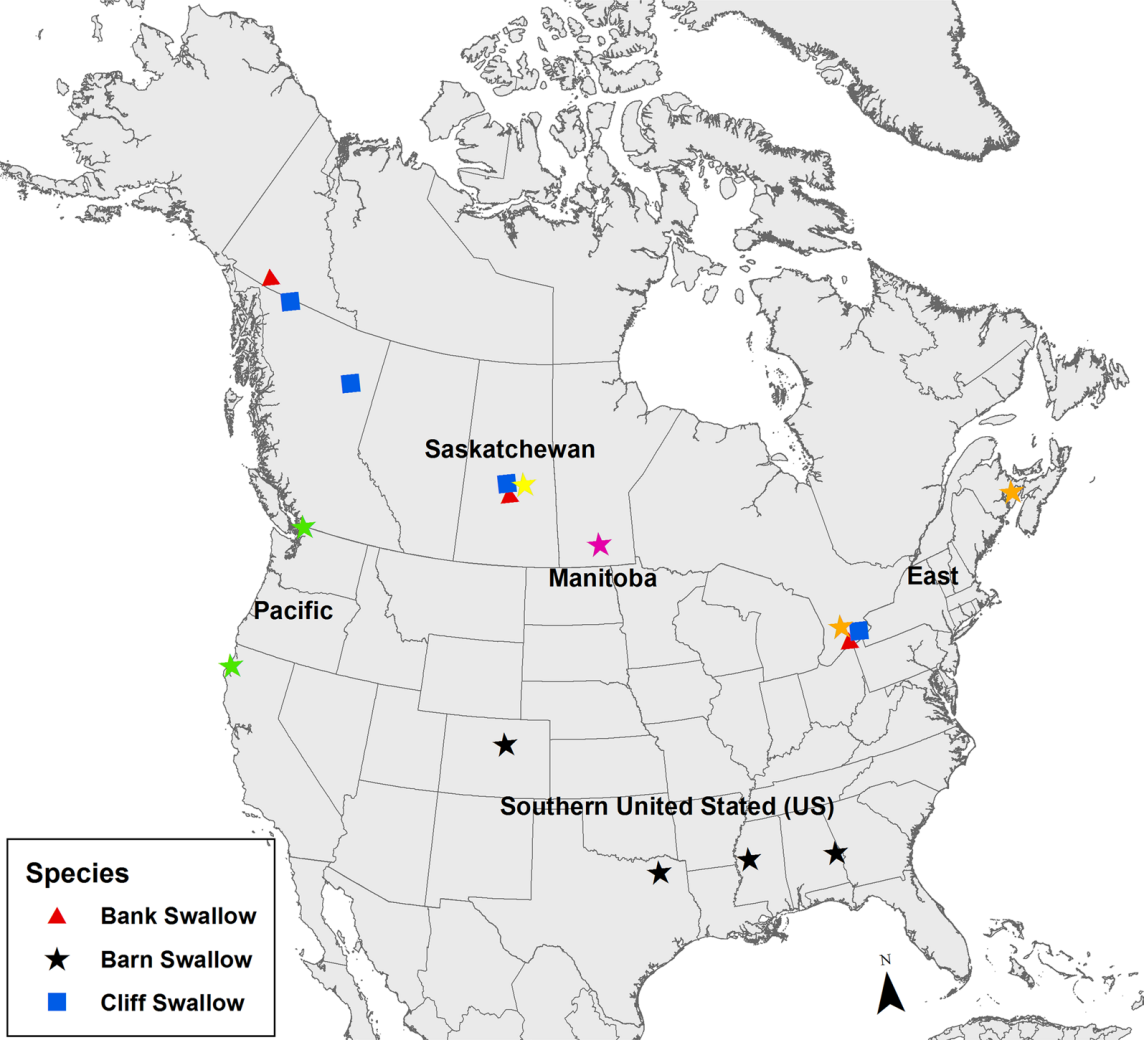
Feather sampling locations from adult Bank, Barn and Cliff Swallow used in this study 2009–2019. Coloured symbols and text for Barn Swallow represent five population groups used in the analyses (see text). Overlapping symbols are offset for clarity

Adult swallows were captured at nesting sites (e.g., riparian banks, barns, bridges) using mist nets at multiple locations in Canada and the United States during the breeding season (June–July), 2009–2019 (Fig. 1). The study was generally designed to investigate migratory connectivity of adult populations across a broad longitudinal range that also spanned the migratory divide identified for Barn Swallow [33] along with a latitudinal gradient across the majority of the North American breeding range of Barn Swallow. Data used in this study were collected for this project or were used in other studies and reanalyzed using different methods or for different purposes [8, 33, 70]. Individuals were aged by plumage characteristics and mouth gape coloration if captured outside the nest, and the sex of adult birds was determined by evidence of cloacal protuberance (male) or brood patch (female). Birds were fitted with a uniquely numbered U.S. Geological Survey metal leg band. We measured unflattened wing chord to the nearest millimeter and plucked one or two inner tail feathers (R3 or R4) from each bird, which we assumed were moulted on the wintering grounds for stable isotope analysis [51, 52].

### Stable isotope analysis

Feathers were stored in paper envelopes and kept dry prior to isotope analysis. All feathers were cleaned of surface oils in a 2:1 chloroform:methanol solvent soak overnight and subsequently rinsed before being dried for 72 h in a fume hood. Samples were assayed for *δ*^2^H, *δ*^13^C, and *δ*^15^N at the Stable Isotope Laboratory of Environment and Climate Change Canada (ECCC), Saskatoon, Saskatchewan, or (*δ*^2^H only) at the University of Western Ontario, London, Ontario. At both laboratories, we determined the nonexchangeable *δ*^2^H value of feathers by the comparative equilibration method using 3 calibrated keratin hydrogen isotope reference materials (CBS: −197%; KHS: −54.1%; SPK: −121.6%). Hydrogen isotopic measurements were performed on 0.35 mg samples loaded into silver capsules and crushed and placed in a zero blank (ECCC) or Uni-Prep carousel (Eurovector, Milan, Italy). H_2_ gas derived from high-temperature (1350 °C) flash pyrolysis in a glassy carbon reactor was introduced into either an interfaced Thermo Delta Plus (Thermo, Bremen, Germany) or an Isoprime (Isoprime, Manchester, UK) continuous-flow isotope-ratio mass spectrometer. Measurement of the keratin laboratory reference materials corrected for linear instrumental drift were both accurate and precise at both laboratories, with typical within-run (n = 5) SD values of 2‰. We report all results for nonexchangeable H using the typical delta (*δ*) notation, in units of per mil (‰) normalized on the Vienna Standard Mean Ocean Water (VSMOW) scale. We consider all of the isotope data used in our study (based on our newly reported data and from previous studies) to be entirely comparable.

Feather samples analyzed for stable carbon (*δ*^13^C) and nitrogen (*δ*^15^N) isotope values were weighed (1.0 mg) in tin capsules, compressed and analyzed at the ECCC stable isotope laboratory in Saskatoon, Saskatchewan, Canada. Samples were combusted at 1030°C in a Carlo Erba NA1500 (Thermo Scientific; Waltham, United States) or Eurovector 3000 (Eurovector, Milan) elemental analyser. The resulting N_2_ and CO_2_ were separated chromatographically and introduced into an Elementar Isoprime (Elementar; Langenselbold, Germany) or a Nu Instruments Horizon (Nu Instruments Ltd.; Wrexham, United Kingdom) isotope ratio mass spectrometer. Sample results were expressed in the standard delta (*δ*) notation in parts per thousand (‰) deviation from international standards (Vienna Pee Dee Belemnite [VPDB] and AIR for *δ*^13^C and *δ*^15^N, respectively). Internal laboratory calibration standards were BWBIII keratin (*δ*^13^C = − 20.18‰, *δ*^15^N = 14.31 ‰) and Pugel (*δ*^13^C = − 13.64‰, δ^15^N = 5.07‰). Measurement precision was based on replicate (n = 5) within-run measurements of internal reference material and estimated to be ± 0.1‰ for both *δ*^13^C and *δ*^15^N.

### Statistical analyses

Populations of Bank and Cliff Swallows sampled for this study on the breeding grounds were > 750 km apart therefore we considered all populations as independent. We initially conducted clustering analyses that included Barn Swallow isotope and wing-length data separately for each sex in order to identify populations with similar migratory behaviors. However, sample sizes of complete datasets of each population were low and so this analysis lacked power to detect population clusters reliably. Our feather isotope dataset for Barn Swallows included individuals from breeding locations in relatively close geographic proximity (Fig. 1) that we expected to share overlapping wintering (i.e. moulting) ranges based on previous research [33] and so we grouped Barn Swallow sampling locations into five populations. We filtered our isotope dataset based on outlier *δ*^***2***^H_f_ values (< − 120‰) because these feathers were likely grown on the breeding grounds (e.g. via feather replacement). Our dataset contained birds captured in multiple years across populations and so we assumed that individuals were representative of non-breeding season use from each population and that the isoscapes did not vary substantially among years. Results of statistical tests were considered significant at *P* < 0.05.

### Assignment to origin

We used a dual-isotope multivariate normal probability density function (mvnpdf) method for assigning birds to origins on the non-breeding grounds (sensu [34, 71]). For each species and population combination, we conducted separate probabilistic assignment to origin analyses using only *δ*^2^H_f_ and *δ*^13^C_f_ and included possible molt origins in Central and South America where these species are expected to spend the non-breeding season for each species and population, separately. We did not use *δ*^15^N_f_ values because of the potential influence of anthropogenic inputs from agriculture that are challenging to model spatially. This multi-isotope mvnpdf approach assumes that the isoscapes are independent and governed by different bioclimatic processes and therefore exhibit spatial non-stationarity (i.e. they were orthogonal).

We converted a growing season precipitation *δ*^2^H (*δ*^2^H_p_) surface [39] to an equivalent feather isoscape using the calibration equation for non ground-foraging Nearctic–Neotropical migrant birds from Hobson et al. [28]: *δ*^2^H_f_ = 17.57 + 0.95 * *δ*^2^H_p_. We used a *δ*^13^C isoscape representing a theoretical spatial distribution of *δ*^13^C values in plants in Central and South America, which is based on annual plant *δ*^13^C composition approximately corresponding to mean annual conditions in the year 2000 and incorporates intra-annual variability in vegetation distribution and productivity. Plant-based isoscapes likely exhibit minimal annual changes in *δ*^13^C and so this isoscape provided the most current and accurate approximation of plant *δ*^13^C composition available for Central and South America. We applied + 2% to the plant *δ*^13^C isoscape to account for discrimination between plants and herbivorous insects in feather isotopes and used this as our feather *δ*^13^C (*δ*^13^C_f_) isoscape (see [72]).

The mvnpdf method estimates the probability that a particular spatially referenced cell could represent a possible origin in a calibrated raster feather isoscape space (*δ*^2^H_f_, *δ*^13^C_f_) as modified from Hobson et al. [71]:

$$fx\left( {x^{i} y^{i} {|}\mu_{{{\text{HC}}}} ,\sigma_{{{\text{HC}}}} ,\rho_{{{\text{HC}}}} } \right) = 2{\uppi }^{ - 1/2k} |\sum |^{ - 1/2k} e^{{[ - 1/2\left( {y - \mu_{x} i} \right)/\sum - 1\left( {y - \mu_{x} i} \right]}}$$

where the spatially explicit probability density function for a possible location (i.e. raster cell) of origin (*x*^*i*^), given an individual feather of unknown origin (*y*^*i*^) having known isotopic (*δ*^2^H_f_, *δ*^13^C_f_) composition, is indicated by *fx*. Subscripted HC represents the predicted mean (µ), standard deviation (σ), and correlation (ρ) of the *δ*^2^H_f_ (H) and *δ*^13^C_f_ (C) values for a feather grown at a particular location (i.e. raster cell), and k indicates the number of isotopes. Mean isotopic composition for a discrete location (*x*^*i*^) was derived from raster cells in the calibrated isoscapes, and µ_*x*_^*i*^ represents a vector of means for individual raster cells. The variance–covariance matrix (|Σ|) of the isoscapes using the 2-isotope environment is expressed as:

$$\sum \, = \left[ {\begin{array}{*{20}l} {\sigma^{2}_{{\delta^{2} {\text{H}}}} } \hfill & {\sigma^{2}_{{\delta^{2} {\text{H}},\delta^{13} {\text{C}}}} } \hfill \\ {\sigma^{2}_{{\delta^{2} {\text{H}},\delta^{13} {\text{C}}}} } \hfill & {\sigma^{2}_{{\delta^{13} {\text{C}}}} } \hfill \\ \end{array} } \right]$$.

We used a conservative odds ratio to assign feathers to potential molt origin using the spatially explicit probability densities for individual samples where georeferenced locations (i.e. raster cells) with ≥ 66.7% probability were coded as potential origins (1) and all other locations (i.e. < 66.7%) were considered as improbable origins (0) [50, 71]. Individual assignment to origin analyses conducted for each sample hence resulted in a spatially referenced binary raster file, which were subsequently summed across assignments for all other individuals to represent potential origins for each species and population grouping.

Unlike Hobson and Kardynal [34], we were unable to use Bayesian prior information from other data to limit assignments or to include as priors such as banding data or geolocation information in the analyses because these data were unavailable or incomplete for multiple populations. We were unable to incorporate wing length into the assignment to origin analyses because we lacked information of spatial gradients on the non-breeding grounds; however, wing length data were incorporated into our niche estimates (below). We used conservative range boundaries for the non-breeding grounds from BirdLife International and NatureServe (2011) in assignment to origin analyses for all species. Assignment to origin analyses including raster and polygon manipulation were conducted using the ‘raster’, ‘rgeos’, ‘mvtnorm’, ‘sp’ and ‘Rfast’ packages in the R v4.1.1 computing environment [73–78].

We used k-means clustering to identify similar spatially contiguous regions of origin based on individual likelihood surfaces for each species, separately. To accomplish this, we first determined the optimal number of clusters in each dataset from the similarity matrix of pairwise probability surfaces bootstrapped 1,000 times resulting in Schoener's D‐metric of spatial similarity in the ‘isocat’ v0.2.6 [59] R package. We then applied hierarchical clustering on the similarity matrices using correlation distances in the ‘pvclust’ v2.2‐0 package [79]. Following preliminary exploration, we cut dendrogram cluster trees at heights of 0.5 which produced 5 clusters for all species and represented a good trade-off between the potential number of population clusters (i.e. too many vs. too few). We then used visualized potential spatially aggregated population clusters based on individual assignment to origin likelihood surfaces using the ‘ecbtools’ v0.2 [80] package. Geographic weighting can be set to values between 1 and 100 to determine clusters where 1 minimizes the importance of distance of each cell from one another and 100 maximizes spatial constraining (i.e. more spatially aggregated clusters). Geographic weighting was set between 10 and 20 with values arbitrarily chosen based on the amount of dispersion of cells from the main cluster. We subsequently determined the maximum likelihood value for each individual across a species-specific cluster defined above using zonal statistics in the raster v3.6-3 [76]. We note that since the cluster analysis is based on the assignments that the analyses are not independent that these should produce similar results; however, clustering provided an additional tool to assess and visualize non-breeding areas.

As a proxy of non-breeding season population dispersion, we estimated overlap of isotopic and isotopic—morphometric niche space among breeding populations within species across populations and among species within breeding populations using isotopes only of adult Bank, Barn and Cliff swallows, separately. This was accomplished using stable isotope ellipses based on Bayesian inference (SIBER; [30]). Here, we compared isotopic and morphometric overlap between a priori derived populations of the three study species with pair-wise isotope combinations (*δ*^13^C_f_ – *δ*^2^H_f_, *δ*^2^H_f_ – *δ*^15^N_f_, *δ*^13^C_f_ – *δ*^2^H_f_), and using all three isotopes and wing length using Bayesian standard ellipse areas (SEA). Inclusion of the three isotopes and/or wing length was completed by first conducting linear discriminant analysis (LDA) and subsequently incorporating the LDA scores of the first two discriminant functions (within-species data only) into niche analyses for each combination of input data, separately. We generated biplots using the above isotope and/or wing length combinations to visualize niche separation and estimated the percentage of niche overlap for the 40% Bayesian ellipses for combinations of species, isotopes, wing length and populations. LDA and niche overlap analyses were conducted using the MASS and SIBER R packages, respectively, in the R 4.1.1 computing environment [30, 78, 81].

### Wing length

We used analysis of variance (ANOVA) to assess differences in wing length for each population × sex combination, separately by species. When the interaction term was significant, we used a subsequent ANOVA to test for differences in wing length among populations separately for males and females. We tested for differences in wing length among populations combined for males and females when the population × sex interaction term was not significant. We then conducted Tukey’s post-hoc test to determine which populations varied significantly. Additional wing length data were accessed from the Canadian Wildlife Service Bird Banding Office and integrated into this analysis.

## Results

### Spatial assignment to isoscapes

**Fig. 2:**
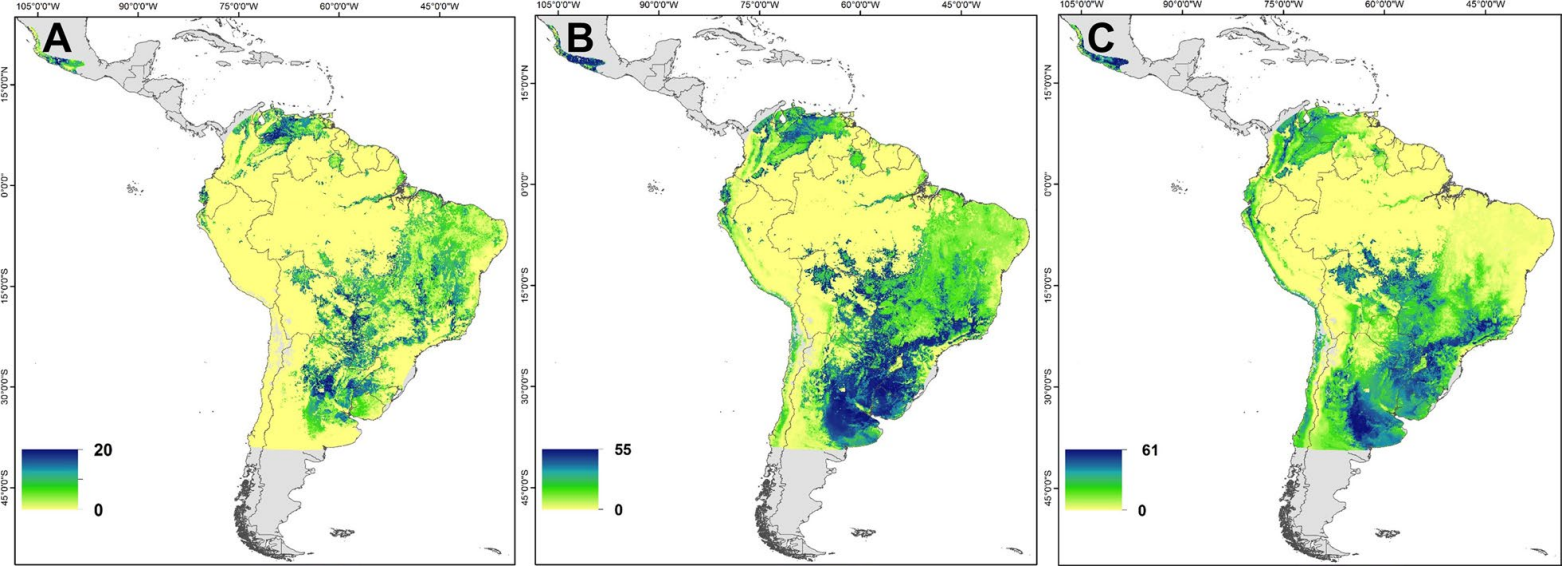
Probabilistic assignment to origins analysis results for Bank Swallows sampled in Yukon (**A**; n = 26), Saskatchewan (**B**; n = 74) and Ontario (**C**; n = 76). The color scale shows the number of samples isotopically consistent with an individual pixel

Multivariate probabilistic assignment to origin analysis based on feather stable isotope (Table 1) measurements identified different potential non-breeding regions among populations within and across species. Regions with high probability of non-breeding origin for Bank Swallows sampled from three breeding populations were in southern Mexico, northern Colombia and Venezuela and/or the southeastern parts of the non-breeding range in Bolivia, Brazil and Argentina (Fig. 2). Individuals from the Yukon breeding population (n = 26) appeared to have a higher likelihood of originating from more localized areas in Venezuela and/or from the Pampas region in southern Brazil to north-central Argentina. Both Saskatchewan (n = 74) and Ontario (n = 76) breeding populations had a higher likelihood of origin from southern Mexico and/or the Pampas region of Argentina, Uruguay and Brazil. Ontario birds had a higher likelihood of more localized origin from central Argentina relative to Saskatchewan birds.

**Table 1:** Mean (± 1SE in per mil) stable isotope values carbon (*δ*^13^C_f_), nitrogen (*δ*^15^N_f_) and hydrogen (*δ*^2^H_f_) of (winter-grown) feathers from adult Bank, Barn and Cliff swallows sampled at breeding locations in Canada and the United States, 2009–2019

Species	Population	N	*δ*^13^C_f_	*δ*^15^N_f_	*δ*^2^H_f_
Bank Swallow	Yukon	26	− 19.75 ± 0.44	10.49 ± 0.2	− 44.05 ± 3.4
	Saskatchewan	74	− 20.15 ± 0.33	10.99 ± 0.13	− 46.61 ± 1.41
	Ontario	76	− 20.41 ± 0.36	10.68 ± 0.16	− 54.07 ± 1.11
Barn Swallow	Pacific group (Pac)	72	− 20.28 ± 0.28	10.34 ± 0.2	− 64.5 ± 1.98
	British Columbia (Pac)	15	− 21.24 ± 0.37	10.88 ± 0.47	− 70.9 ± 3.68
	Seattle (Pac)	38	− 19.78 ± 0.46	10.25 ± 0.3	− 65.23 ± 3.17
	California (Pac)	19	− 20.53 ± 0.39	10.09 ± 0.28	− 57.99 ± 2.24
	Saskatchewan	49	− 21.89 ± 0.39	10.31 ± 0.15	− 71.31 ± 2.43
	Manitoba	43	− 20.28 ± 0.49	10.16 ± 0.17	− 62.36 ± 1.68
	East group	220	− 17.69 ± 0.25	11.32 ± 0.11	− 50.13 ± 0.94
	Ontario (East)	167	− 17.81 ± 0.29	11.3 ± 0.12	− 49.36 ± 1.09
	Quebec (East)	10	− 16.03 ± 0.76	11.89 ± 0.53	− 52.41 ± 3.95
	New Brunswick (East)	43	− 17.61 ± 0.61	11.26 ± 0.27	− 52.6 ± 2.09
	S. United States (sUS)	140	− 18.49 ± 0.32	11.13 ± 0.13	− 44.66 ± 1.07
	Colorado (sUS)	25	− 19.75 ± 0.6	10.66 ± 0.29	− 47.09 ± 2.57
	Texas (sUS)	31	− 18.82 ± 0.73	10.96 ± 0.29	− 47.01 ± 2.04
	Mississippi (sUS)	33	− 17.82 ± 0.65	11.45 ± 0.25	− 36.97 ± 1.7
	Alabama (sUS)	51	− 18.12 ± 0.56	11.26 ± 0.22	− 47.03 ± 1.86
Cliff Swallow	Yukon	59	− 20.73 ± 0.19	12.12 ± 0.11	− 51.73 ± 0.87
	British Columbia	14	− 19.54 ± 0.27	12.05 ± 0.26	− 57.94 ± 1.38
	Saskatchewan	55	− 20.42 ± 0.2	12.2 ± 0.09	− 51.63 ± 0.75
	Ontario	37	− 16.65 ± 0.35	11.42 ± 0.14	− 49.48 ± 1.35

**Fig. 3:**
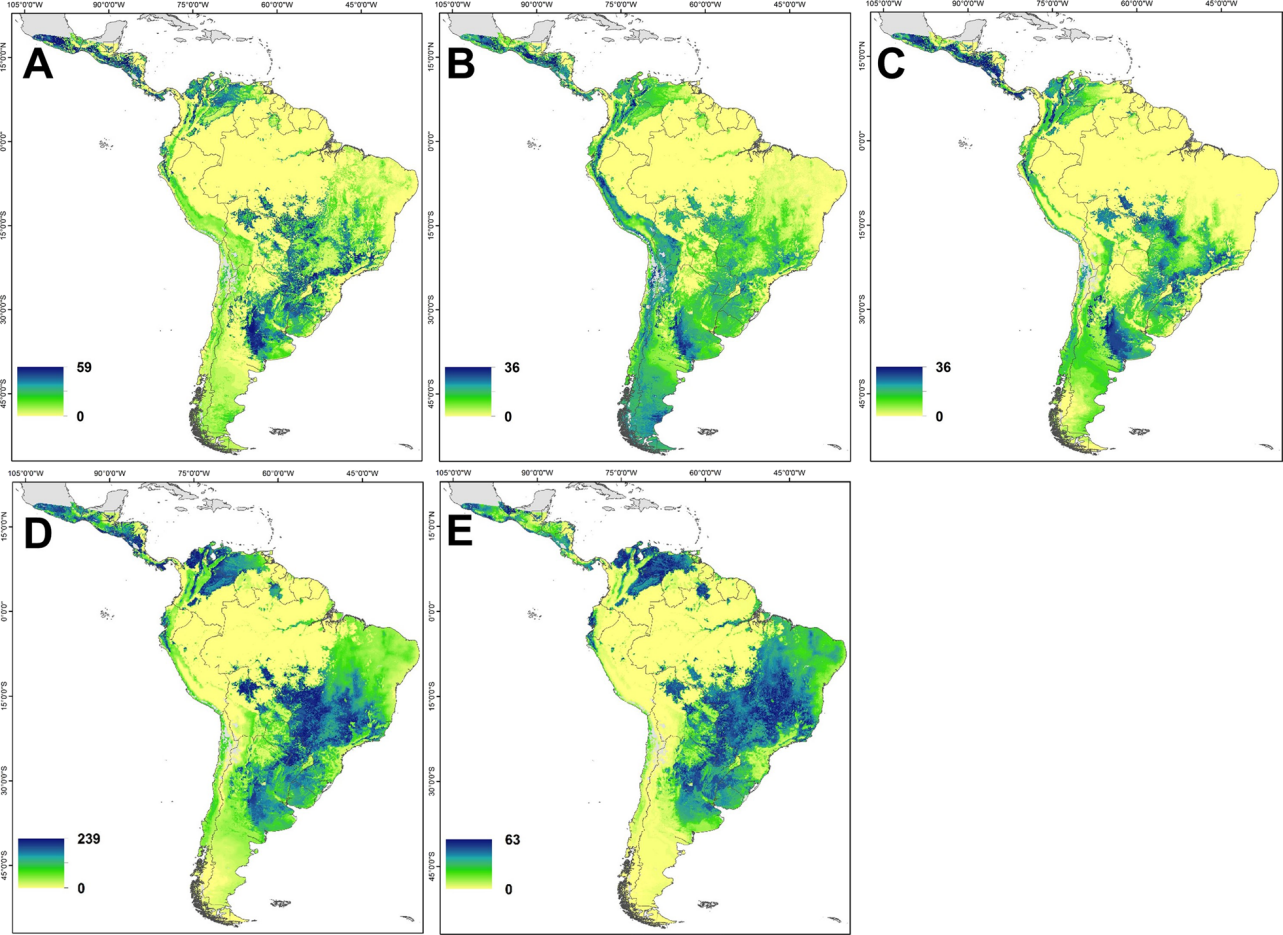
Predicted non-breeding (wintering) origins of North American Barn Swallows sampled from breeding populations in the Pacific (**A**; British Columbia, Washington, California; n = 72), Saskatchewan (**B**; n = 49), Manitoba (**C**; n = 43), eastern Canada (**D**; Ontario, New Brunswick; n = 529), and the southern United States (**E**; Colorado, Texas, Mississippi, Alabama; n = 140) determined using a geospatial assignment based on a multivariate normal probability density function using feather *δ*^13^C and *δ*^2^H assays. The color scale shows the number of samples isotopically consistent with an individual pixel

Likely non-breeding regions of origin for the five populations of Barn Swallows were distributed across wide parts of Central and South America (Fig. 3). Central America was a potential region of origin for Barn Swallows sampled on the Pacific Coast (n = 72), Saskatchewan (n = 49), Manitoba (n = 43), and eastern Canada (n = 522) but not for birds sampled in the southern United States (n = 140). Central Argentina had a high likelihood of provenance for Barn Swallows from the three western populations. The three western populations of Barn Swallow also had a higher likelihood of origin from along the west Coast of South America including Colombia, Ecuador and Peru relative to the other sampled populations. Northern Colombia and Venezuela and/or a large region from southern Brazil and northern Bolivia to the Pampas of Argentina was also a potential region of origin for birds sampled in Manitoba, eastern Canada and the southern United States.

**Fig. 4:**
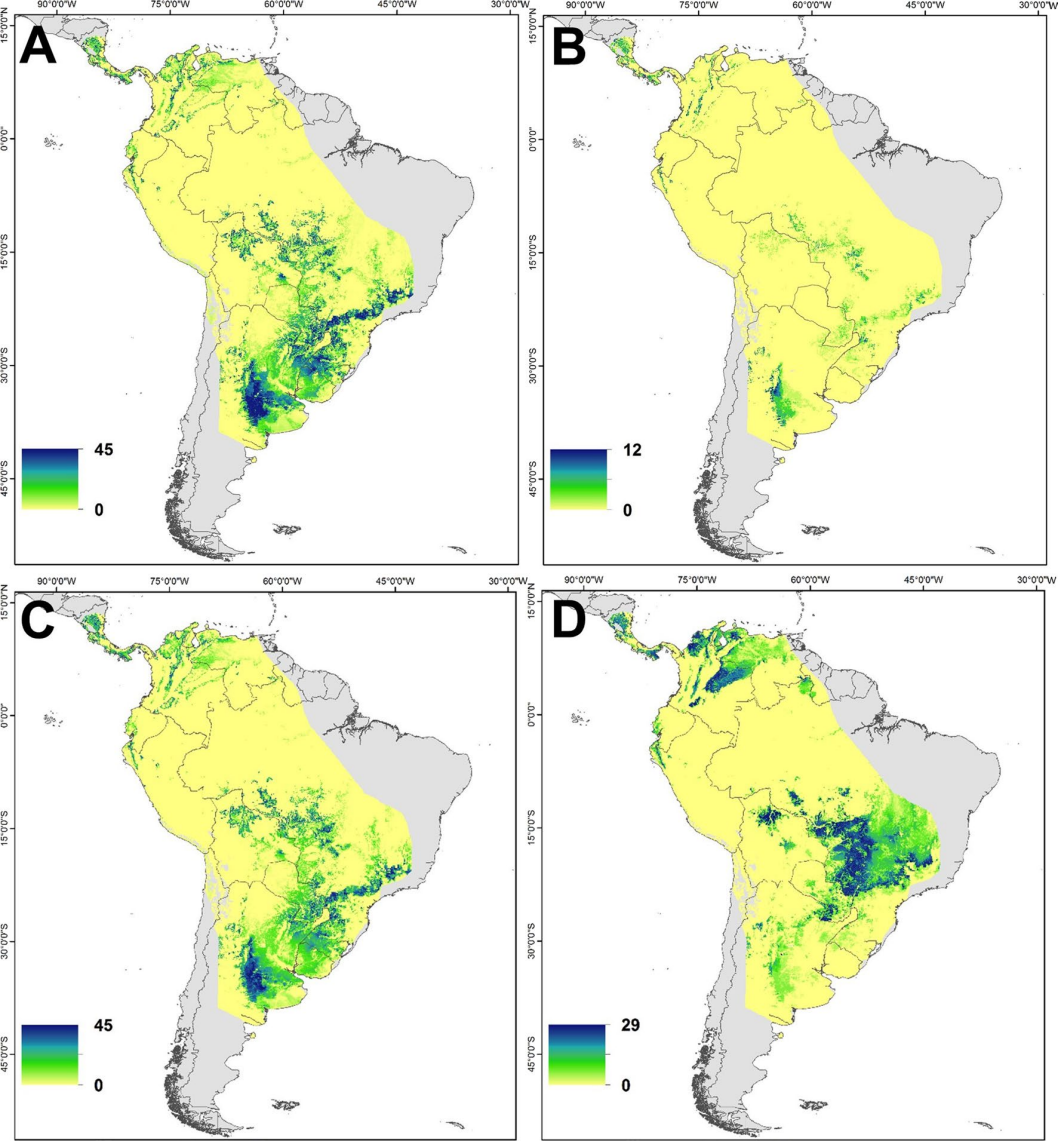
Predicted non-breeding origins (wintering) of Cliff Swallows sampled from breeding populations in southern Yukon (**A**; n = 59), central British Columbia (**B**; n = 14), central Saskatchewan (**C**; n = 55) and southern Ontario (**D**; n = 55) determined using a geospatial assignment based on a multivariate normal probability density function using feather *δ*^13^C and *δ*^2^H assays. The color scale shows the number of samples isotopically consistent with an individual pixel

For Cliff Swallows, Central Argentina and Pampas region of Uruguay and Brazil had a high likelihood of origin from the southern Yukon (n = 59), central British Colombia (n = 14) and Saskatchewan (n = 55; Fig. 4). Most probable origins of Cliff Swallows sampled in southern Ontario (n = 55) were west central Colombia and central Brazil, the latter being farther north than the other sampled populations. All populations of the three species of swallow appeared to avoid the Amazon Basin except for the lower reaches of the Amazon River from which some Bank and Barn Swallows potentially originated.

### Spatial clustering

**Fig. 5:**
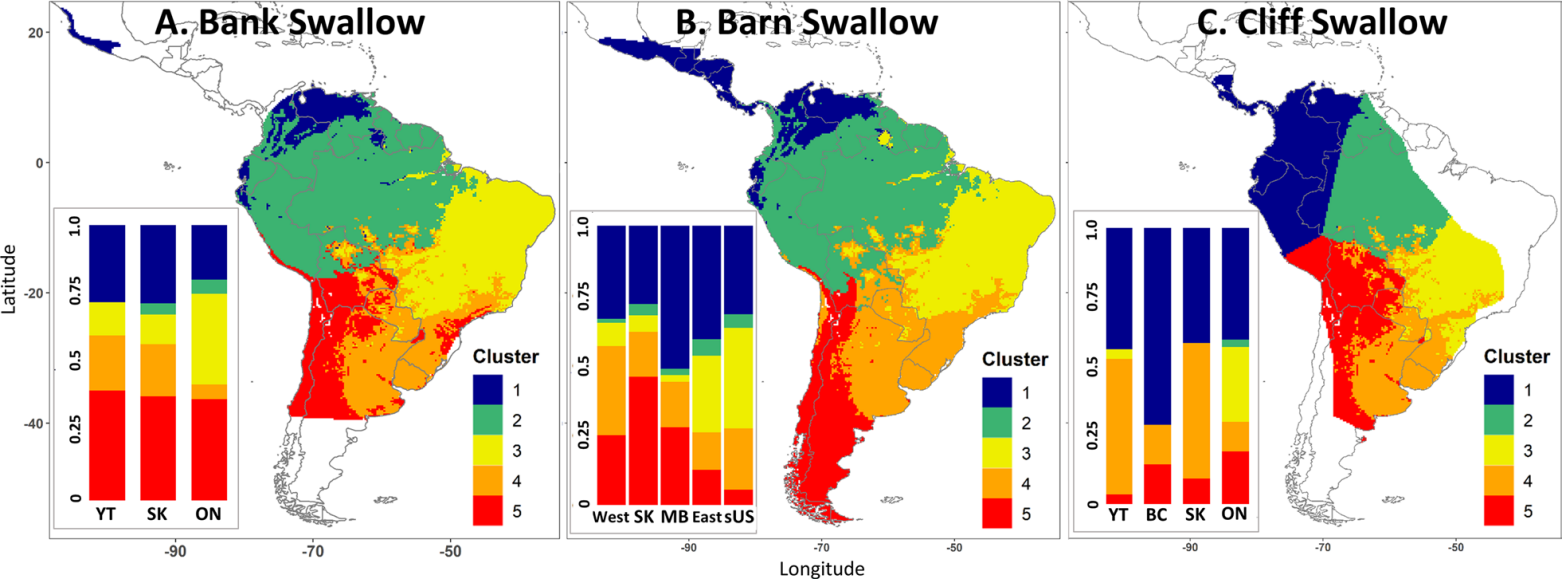
Clusters representing potential areas of non-breeding origin for **A** Bank, **B**, Barn, and **C** Cliff Swallow from birds captured on the North American breeding grounds from k-means clustering and assigned to Latin American non-breeding grounds. Location codes for breeding populations in bar graph: YT—Yukon Territory, BC—British Columbia, West—Western Canada and United States, SK—Saskatchewan, MB—Manitoba, ON—Ontario, East—eastern Canada, sUS—southern United States; see Fig. 1. Barplots represent the proportion of individuals from each breeding population likely originating from each spatial isotopic cluster using zonal statistics

Spatial clustering of individual assignment surfaces based on both stable isotope measurements (*δ*^2^H, *δ*^13^C) of feathers identified five main probable regions of origin in Latin America for all species of swallow analyzed separately when the cluster dendrogram was cut at the 0.5 level (Fig. 5). Regions encompassed within clusters were generally consistent across species with some spatial variation: cluster 1—Central America and northern South America, cluster 2—Amazon Basin, cluster 3—eastern South America (mostly eastern and southern Brazil), cluster 4—east Central South America, and cluster 5—southern and western South America. Clusters for Cliff Swallow varied slightly from the other two species with cluster 1 encompassing more of northern South America and cluster 5 including more of the central part of the continent. Across populations, Bank Swallows apparently originated from clusters 1 and 5 with more Ontario-breeding individuals originating from cluster 3 than the other two populations (Fig. 5A). Consistent with the probabilistic assignments, populations of Barn Swallows that bred in western North America likely originated from clusters 1, 5 and 4 with more individuals from Saskatchewan apparently having provenance from cluster 5 than the other two western breeding populations (Fig. 5B). Most Barn Swallows breeding in eastern Canada and the southern United States apparently spent the non-breeding season in clusters 1 and 3. Cliff Swallows showed greater variation in cluster origin among populations with breeding individuals from Yukon and Saskatchewan mostly originating from cluster 1 and 4, a greater proportion of individuals from British Columbia originating from cluster 1, and more Ontario breeders spending the non-breeding season in clusters 1 and 3 (Fig. 5C).

### Isotopic niche overlap

**Fig. 6:**
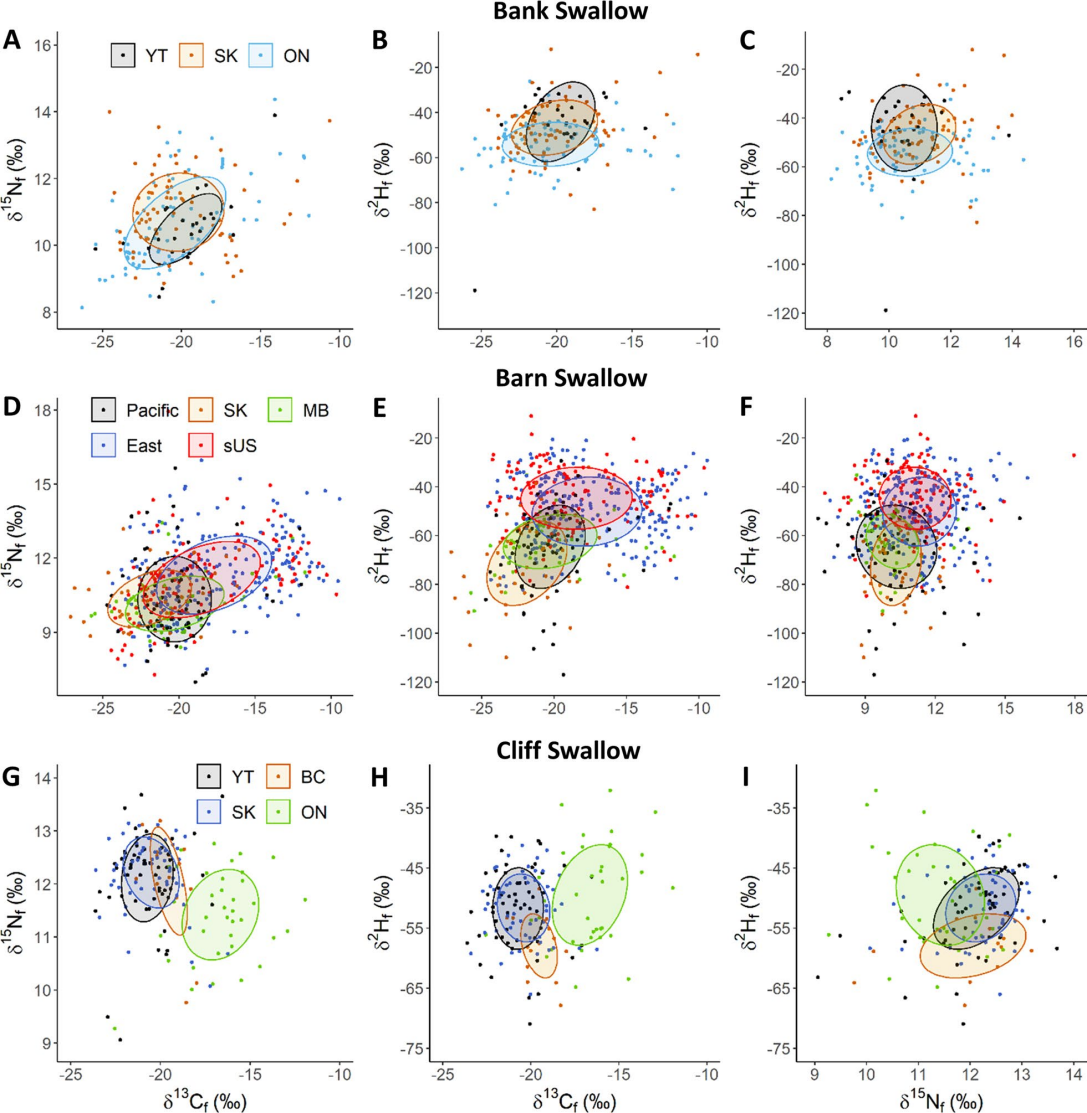
Biplots with 40% Bayesian ellipses showing feather isotopic overlap in *δ*^13^C–*δ*^2^H, *δ*^13^C–*δ*^15^N, and *δ*^15^N–*δ*^2^H for adult Bank (**A**–**C**), Barn (**D**–**F**) and Cliff (**G**–**I**) swallows sampled in Canada and the United States. Feather isotope values represent non-breeding ground (winter) molt locations. Increasing distance between ellipses indicates greater isotopic dissimilarity. Sampling locations: YT—Yukon, BC—British Columbia, SK—Saskatchewan, ON—Ontario; sampling locations grouped for Barn Swallows: Pacific: BC, Washington, California; SK; MB—Manitoba; East—Ontario, New Brunswick; sUS—southern United States (Colorado, Texas, Mississippi, Alabama). Niche area and overlap are in Table 1 and biplots showing isotopic and wing length overlap are presented in Additional file 1: Figure S2 and Table S3

Comparisons among populations across species showed variable isotopic and/or morphometric niche overlap with generally greater overlap within breeding regions. Biplots contrasting feather isotope (*δ*^2^H, *δ*^13^C, *δ*^15^N) and wing lengths of conspecific breeding swallows generally indicated little separation in the 40% Bayesian ellipses for Bank Swallows among three widely separated Canadian breeding populations but highest separation between Yukon and Ontario populations, including when wing length was included in the analyses (two isotope comparisons: 8.2–42.0% overlap; three isotope and wing length: 17.3–32.2%; Fig. 6A–C; Tables 2, 3 see also S2 and S3). Barn Swallows occupied consistently highest niche space among breeding populations with substantial separation between western (Pacific, Saskatchewan) and eastern Canadian (4.0–25.6%) and southern U.S. breeding populations (0.8–39.6%) with considerable overlap of Manitoba birds with both western and eastern populations (11.8–61.1%; Fig. 6D–F). Incorporation of wing length appeared to increase niche separation based solely on isotope data of Barn Swallows between Pacific and eastern populations (from ~ 14.8 to 0.5% overlap), Manitoban with western populations (61.1% to 25.8% overlap), and eastern and southern U.S. populations (54.5% to 28.8% overlap). Biplots for Cliff Swallows showed high niche separation between western (Yukon, British Columbia, Saskatchewan) and eastern (Ontario) populations (0–28.3% overlap) and low niche differentiation between Yukon and Saskatchewan populations (67.0 to 71.6% overlap) with greater niche separation between those two populations and central British Columbia birds (0–35.1% overlap; Fig. 6G–I).

**Table 2:** Area and overlap of maximum likelihood fitted 40% Bayesian ellipses estimated using standard ellipse areas assessed on combinations of feather isotope (*δ*^13^C_f_, *δ*^15^N_f_, *δ*^2^H_f_) and wing length of multiple populations of Bank, Barn and Cliff swallows sampled in Canada and the United States

Species	Isotope	Population	Area 1 (‰)^2^	Area 2 (‰)^2^	Overlap (%)
Bank Swallow (N = 175)	*δ*^2^H_f_, *δ*^13^C_f_	YT—SK	53.8	107.5	43.2
		YT—ON	53.8	99.4	8.2
		SK—ON	107.5	99.4	40.1
	*δ*^13^C_f_,* δ*^15^N_f_	YT—SK	5.1	10.6	39.1
		YT—ON	5.1	11.3	42.0
		SK—ON	10.6	11.3	63.9
	*δ*^15^N_f_, *δ*^2^H_f_	YT—SK	29.0	44.4	34.3
		YT—ON	29.0	42.7	9.7
		SK—ON	44.4	42.7	24.3
Barn Swallow (N = 522)	*δ*^2^H_f_,* δ*^13^C_f_	Pacific—SK	126.1	155.8	40.2
		Pacific—MB	126.1	111.6	61.1
		Pacific—East	126.1	168.8	17.8
		Pacific—s. U.S	126.1	155.2	10.0
		SK—MB	155.8	111.6	36.2
		SK—East	155.8	168.8	4.0
		SK—s. U.S	155.8	155.2	0.8
		MB—East	111.6	168.8	18.3
		MB—s. U.S	111.6	155.2	7.5
		East—s. U.S	168.8	155.2	53.5
	*δ*^13^C_f_, *δ*^15^N_f_	Pacific—SK	13.3	9.3	36.0
		Pacific—MB	13.3	10.8	58.0
		Pacific—East	13.3	17.2	25.6
		Pacific—s. U.S	13.3	16.7	39.6
		SK—MB	9.3	10.8	40.1
		SK—East	9.3	17.2	12.0
		SK—s. U.S	9.3	16.7	21.6
		MB—East	10.8	17.2	23.7
		MB—s. U.S	10.8	16.7	39.4
		East—s. U.S	17.2	16.7	73.9
	*δ*^15^Nf, *δ*^2^H_f_	Pacific—SK	93.1	69.8	47.9
		Pacific—MB	93.1	39.1	42.0
		Pacific—East	93.1	68.9	19.3
		Pacific—s. U.S	93.1	62.2	9.2
		SK—MB	69.8	39.1	40.0
		SK—East	69.8	68.9	7.8
		SK—s. U.S	69.8	62.2	1.4
		MB—East	39.1	68.9	11.8
		MB—s. U.S	39.1	62.2	3.1
		East—s. U.S	68.9	62.2	55.7
Cliff Swallow (N = 165)	*δ*^13^C_f_ *δ*^15^Nf	YT—BC	3.8	2.8	24.5
		YT—SK	3.8	3.1	67.0
		YT—ON	3.8	5.7	0.0
		SK—ON	3.1	5.7	0.0
		BC—ON	2.8	5.7	1.6
		SK—BC	3.1	2.8	35.1
	*δ*^2^H_f_, *δ*^15^Nf	YT—BC	31.1	17.0	11.7
		YT—SK	31.1	26.7	70.2
		YT—ON	31.1	54.7	0.0
		SK—ON	17.0	26.7	14.4
		BC—ON	17.0	54.7	0.0
		SK—BC	26.7	54.7	0.0
	*δ*^15^N_f_, *δ*^2^H_f_	YT—BC	16.1	16.9	21.0
		YT—SK	16.1	11.9	71.6
		YT—ON	16.1	22.5	28.3
		BC—SK	16.9	11.9	17.0
		BC—ON	16.9	22.5	9.3
		SK—ON	11.9	22.5	20.9

Feather isotope values represent non–breeding ground (winter) molt locations. Higher overlap indicates greater isotopic and/or wing length similarity and apparent lower migratory connectivity. Sampling locations: YT—Yukon, BC—British Columbia, SK—Saskatchewan, ON—Ontario; sampling locations grouped for Barn Swallows: Pacific – BC, Washington, California, SK, MB—Manitoba, East—ON, New Brunswick, sUS—southern United States (Colorado, Texas, Mississippi, Alabama). Biplots showing isotopic and wing length overlap are presented in Fig. 6 and Additional file 1: Figure S2

**Table 3:** Area (per mil)^2^ and overlap of maximum likelihood fitted 40% Bayesian ellipses estimated using standard ellipse areas assessed on combinations of feather isotopes (*δ*^13^C_f_, *δ*^15^N_f_, *δ*^2^H_f_) of populations of Bank (BANS), Barn (BARS) and Cliff (CLSW) swallows sampled in Yukon, SK and ON, Canada

Population	Isotope Comparison	Species (Area 1–Area 2)	Area 1	Area 2	Overlap (%)
SK	*δ*^13^C_f_, *δ*^15^N_f_	BANS—BARS	11.1	9.2	29.0
		BANS—CLSW	11.1	3.5	12.7
		BARS—CLSW	9.2	3.5	0.0
	*δ*^13^C_f_, *δ*^2^H_f_	BANS—BARS	136.7	155.8	9.6
		BANS—CLSW	136.7	71.1	40.7
		BARS—CLSW	155.8	71.1	17.2
	*δ*^15^N_f_, * δ*^2^H_f_	BANS—BARS	57.5	69.8	0.0
		BANS—CLSW	57.5	31.6	10.0
		BARS—CLSW	69.8	31.6	0.0
ON	*δ*^13^C_f_, * δ*^15^N_f_	BANS—BARS	11.3	16.9	32.3
		BANS—CLSW	11.3	5.7	10.1
		BARS—CLSW	16.9	5.7	33.8
	*δ*^13^C_f_, * δ*^2^H_f_	BANS—BARS	99.4	166.9	29.2
		BANS—CLSW	99.4	54.7	9.5
		BARS—CLSW	166.9	54.7	32.8
	*δ*^15^N_f_, * δ*^2^H_f_	BANS—BARS	42.7	68.5	43.5
		BANS—CLSW	42.7	22.5	30.8
		BARS—CLSW	68.5	22.5	32.9
Yukon	*δ*^13^C_f_, * δ*^15^N_f_	BANS—CLSW	6.0	3.8	0.0
	*δ*^13^C_f_, * δ*^2^H_f_	BANS—CLSW	120.2	31.1	25.3
	*δ*^15^N_f_, * δ*^2^H_f_	BANS—CLSW	60.4	16.1	0.5

Feather isotope values represent non–breeding ground (winter) molt locations. Higher overlap indicates greater isotopic similarity and supposed lower migratory connectivity

**Fig. 7:**
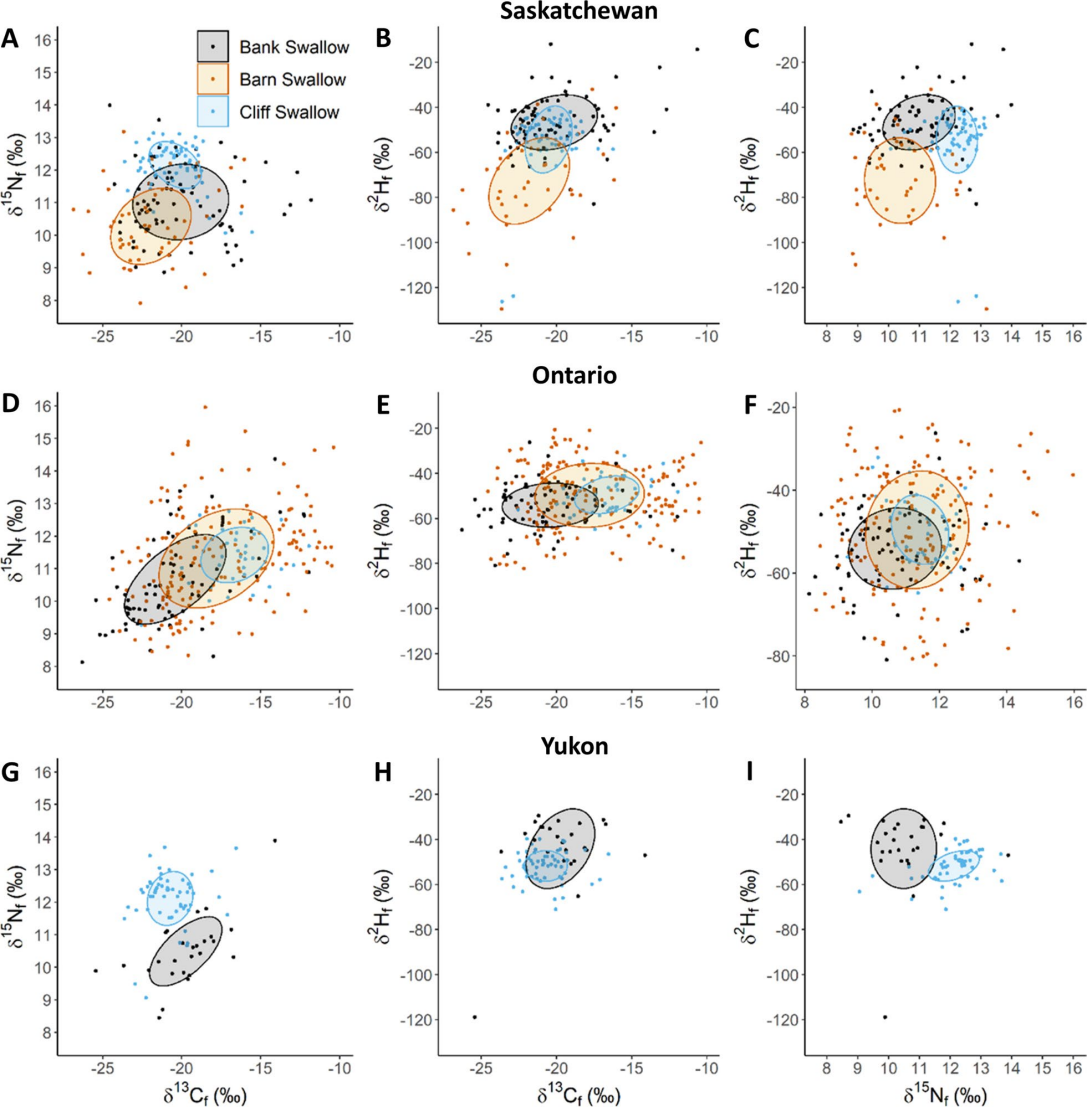
Biplots with 40% Bayesian ellipses showing feather isotopic overlap in *δ*^13^C–*δ*^2^H, *δ*^13^C–*δ*^15^N, and *δ*^15^N–*δ*^2^H for adult swallows from breeding populations in Saskatchewan (**A**–**C**), Ontario (**D**–**F**) and Yukon (**G**–**I**). Feather isotope values represent non-breeding ground (winter) molt locations. Barn Swallows reliably showed the highest isotopic values consistent with the most terrestrial diet. Increasing distance between ellipses indicates greater isotopic dissimilarity

Contrasts among species within similar breeding locations showed moderate overlap of 40% Bayesian ellipses with *δ*^13^C_f_ and *δ*^15^N_f_ for Bank Swallows with Barn (29.0%) and Cliff Swallows (12.7%) and no overlap between Barn and Cliff Swallows in Saskatchewan (Fig. 7A). Saskatchewan Bank and Cliff Swallow *δ*^13^C_f_ and *δ*^2^H_f_ Bayesian Ellipses had low to moderate overlap and both these species had lower overlap with Barn Swallow (9.6 and 40.7%, respectively; Fig. 7B). The *δ*^15^N_f_ and *δ*^2^H_f_ 40% Bayesian ellipses did not overlap for Bank Swallow with Barn (0%) and minimally with Cliff Swallow (10%) but not for the latter two species (Fig. 7C). In Ontario, the three species of swallow had low to high overlap for all isotope biplot 40% Bayesian ellipses but with generally less overlap of Bank and Cliff Swallow ellipses (9.5 to 30.8%) and high overlap for Barn and Cliff Swallow ellipses (32.8 to 33.8%; Fig. 7D–F). Bayesian ellipses for the two species we sampled in the Yukon had no or minimal overlap for *δ*^13^C_f_–*δ*^15^N_f_ (0%) and *δ*^15^N_f_ and *δ*^2^H_f_ (0.5%) biplots, respectively, but moderate overlap for *δ*^13^C_f_ and *δ*^2^H_f_ biplots (25.3%; Fig. 7G–I).

### Wing length

The ANOVA testing for differences in Bank Swallow wing length among sexes and populations was not significant (*p* = 0.21) but wing length (combined males and females) did vary significantly among populations (*p* < 0.001; Additional file 1: Fig. S1). The Tukey post-hoc test indicated significant differences in wing length between populations in Yukon and Ontario (*p* = 0.003), and Saskatchewan and Ontario (*p* = 0.008) with comparisons between Yukon and Saskatchewan being non-significant (all *p* > 0.26). Similarly, the ANOVA contrasting wing length among sexes and populations for Barn Swallow was not significant (*p* = 0.71) and Tukey post-hoc tests indicated significant differences in wing length between sexes only in New Brunswick with males having longer wings than females (*p* < 0.001). When female and males were combined, birds from Washington had significantly shorter wings than birds from Saskatchewan (*p* < 0.001), Ontario (*p* < 0.001), Quebec (*p* < 0.05), New Brunswick (*p* < 0.001), Texas (*p* < 0.05), Mississippi (*p* = 0.002) and Alabama (*p* < 0.001). Saskatchewan Barn Swallows had shorter wings than New Brunswick (*p* < 0.001), Manitoba birds had shorter wings than Ontario (*p* = 0.004) and New Brunswick (*p* < 0.001), and New Brunswick birds had longer wings than bird from Ontario, Ohio, Texas, Mississippi and Alabama (all *p* < 0.001). For Cliff Swallow, the ANOVA testing for wing length differences across populations and sexes was not significant (*p* = 0.23); however, males had significantly longer wings than females in Ontario. Post-hoc contrasts among populations indicated that Yukon Cliff Swallow wings were longer compared to all other sampled populations (all *p* < 0.01), and British Columbia (*p* < 0.001) and Saskatchewan (*p* < 0.001) having significantly longer wings than Ontario birds.

## Discussion

Using a combination of analytical methods, we contrasted, for the first time, likely non-breeding origins and migratory connectivity of multiple breeding populations of Bank, Barn and Cliff swallow sampled across North America. Using feather stable isotopes (*δ*^2^H_f_, *δ*^13^C_f_,), swallows were probabilistically assigned to Central and South American *δ*^2^H_f_ and *δ*^13^C_f_ isoscapes and subsequently used in cluster analysis to define broad species and population-specific provenance. We then investigated patterns of multi-isotopic (*δ*^2^H_f_, *δ*^13^C_f_, *δ*^15^N_f_) and morphometric (i.e. wing length) niche occupancy among populations within and across species by quantifying niche size and overlap among isotopic and isotopic—morphometric biplots under the assumption that broad and overlapping niches were consistent with low within-species connectivity and narrow niches with higher connectivity. We predicted higher among-population connectivity for colonial breeding Bank and Cliff swallow vs. more solitary nesting Barn Swallows. In general, we found low migratory connectivity for all three species of swallow although evidence was found for a large-scale continental divide in North America and differences in degree of connectivity (i.e. niche overlap) between solitary nesting Barn Swallows and the more colonial Cliff and Bank swallows. While it is clear that using stable isotopes to establish patterns of migratory connectivity can involve considerable spatial ambiguity in assignments, a result of the underlying nature of prevailing isoscapes, we argue that the approach can provide a rapid and convenient means of establishing minimum differences in migratory destinations among groups of species or individuals. This information then allows hypotheses to be generated that can be tested with other tools (e.g. genetics, bio-tracking devices).

### Assignment using isoscapes

The assignment to origin, clustering and isotopic niche analyses of Barn Swallows indicated separation of western breeding populations from both eastern Canadian and southern U.S. populations aligning with a potential migratory divide found using solar geolocators for this species [33]. However, relatively high overlap of 40% Bayesian ellipses for eastern and southern Barn Swallow populations contrasted with earlier preliminary results from the Hobson, Kardynal [33] study, which showed potential over-wintering in northern South America for the southern U.S. population also consistent with retrieval of a single geolocator. These results suggest southern U.S. breeding Barn Swallows may also be travelling to southern Brazil and northern Argentina to winter as predicted for eastern Canadian breeding populations; however, northern Colombia and western  Venezuela also had similar likelihoods of origin due to similarities in underlying isoscape values.

Cliff Swallows also showed variation in niche analyses between western and eastern populations potentially indicating a similar migratory divide for this species occurring approximately between Saskatchewan and Ontario similar to that demonstrated for Barn Swallows. Likely non-breeding areas for Cliff Swallows in southeastern Brazil from the eastern breeding population (i.e. Ontario) had similar origins to Cliff Swallows fit with geolocators in New Brunswick in eastern Canada [35]. However, probabilistic assignment to origin analyses of Cliff Swallow using *δ*^2^H_f_ and *δ*^13^C_f_ in the Imlay et al. [35] study showed closer alignment of non-breeding origins with our western Cliff Swallow populations. Within western breeding populations, Cliff Swallows from British Columbia had lower niche overlap with the Yukon and Saskatchewan populations suggesting different non-breeding areas for these birds. Bank swallows showed the greatest niche overlap suggesting an overall similar wintering region or insufficient information to infer migratory connectivity differences using probabilistic isotopic assignment alone.

Interestingly, our study provided weak evidence that more colonial breeding species (i.e. Cliff and Bank Swallows) had a greater probability of strong migratory connectivity (through narrower niche area) than the more solitary Barn Swallow. Possibly, behavioural mechanisms associated with colonial breeding also operate during migration and wintering. It would be useful to test this hypothesis on other species and populations. On the other hand, swallows are well known for forming large communal foraging and roosting aggregations in wetlands during migration and on the wintering grounds [67, 69, 82] and so even solitary nesting species like Barn Swallow may exhibit greater association with conspecifics and heterospecifics when not breeding.

We found evidence for differences in wing length for all three swallow species associated with breeding origins with generally longer wings for Bank and Cliff Swallow from populations in the north and west, and longer wings in northeastern (i.e. New Brunswick) Barn Swallow populations. While our sample sizes and sampling distributions were low for some regions and species, spatial trends in wing length potentially indicate differences in migration distance for each population where longer wings are typically associated with greater distance [60, 83]. Although not significant for most populations, male wing lengths generally trended longer than females potentially suggesting sex-specific differences in over-wintering areas or simply indicating differences in body size. We integrated wing lengths into our niche analysis but were unable to use those data in combination with isotope data to spatially cluster populations because of a lack of captures from the non-breeding grounds; however, future studies may be able to integrate multiple types of data to further refine spatial assignments to origin. We suspect that integration of multiple markers including wing length will ultimately improve our ability to accurately determine origins of birds to breeding and non-breeding grounds [34, 84–86].

### Using isotopic niches in migratory connectivity research

Ideally, the percent overlap of isotopic niches between populations of interest could be used to quantify minimal migratory connectivity. The problem with this approach, of course, is the fact that isoscapes (and often morphometric gradients) are typically not defined well enough to understand how overlap per se constitutes actual spatial segregation of populations. There are exceptions and authors have previously even used advanced statistical methods to forensically evaluate likely pathways taken by animals in both terrestrial [87, 88] and marine systems [89] based on sequential *δ*^2^H, strontium *δ*^87^Sr, or *δ*^13^C measurements. Those studies were based on association of stable isotope ratios in tissues that preserve temporal records through growth (e.g. claws, baleen, tusk) together with detailed high-resolution isoscapes (see also 88) and have the advantage of not needing to consider among-individual isotopic variance. Of more general use for investigating patterns of connectivity will be the quantification of the percent non-overlap of niches since we have much more confidence that such non-overlap directly implies real spatial segregation whereas overlap among individuals can be ambiguous. This is why we have used the term “minimal” connectivity. Future applications of this approach may therefore be much more suited to well-constrained isotopic systems and species with well-defined diets, habitat use and morphometric gradients on the wintering grounds. In our case, the assumption that swallows had similar (aerial insectivorous) diets and occupied more riparian and wetland-associated regions on the wintering grounds is reasonable.

Understandably, the concept of isotopic niche as a proxy for ecological niche in animals has come under considerable scrutiny [31]. Limitations to this approach include the fact that the isotopic niche is influenced by several factors in addition to diet or trophic position. Some of these limitations can be overcome by incorporating several niche axes including the use of non-isotopic information that better represent the ecology of animals being considered. For example, fatty acid compositional analyses can provide additional niche dimensions [90] and there is considerable scope for combining several other measurements including tissue contaminant profiles providing all metrics are expressed as probabilities or are dimensionless (see [91, 92]). Other authors have suggested that niche width and overlap calculations must consider non-regular geometries (i.e. non ellipsoidal) or those containing natural “holes” not occupied by sampled populations [93, 94]. Advances in statistical approaches that allow for alternate niche geometries and the use of sensitivity analyses to evaluate sampling issues are expected to refine some of these limitations in the future. Our consideration here is primarily the use of isotopic niche overlap as a means to address spatial differences in locations of tissue synthesis vs. ecological niche per se but such refinements will undoubtedly also benefit future use of stable isotope approaches to investigate migratory connectivity. Certainly, in our case, the use of 40% Bayesian ellipses to investigate area of niche overlap for the three biplots involving isotopic data can be considered highly conservative. Possibly, a more realistic approach would be to consider the total dimensional space occupied by all individuals since those occurring outside of the core 40% ellipse represent those individuals more likely to be moulting in different non-breeding regions. Combining all data into ellipsoid volume might also be more realistic depending on the isotopes being considered [91] but the biplot approach avoids some of the issues of interpretation of multivariate data (e.g. [95]). Indeed, *δ*^2^H (and to a lesser degree *δ*^18^O) measurements are expected to provide much more geographic information compared to *δ*^13^C and especially *δ*^15^N measurements arguing for inspection of isotopic biplot areas vs multiple isotopic volumes or hyper volumes.

The argument can be made that the best isotopic approach to considering migratory connectivity is through the probabilistic assignment of individuals and populations to isoscapes rather than through the broader concept of isotopic niche. Regardless of whether or not the isotope niche approach can better inform migratory connectivity patterns, such isotopic analyses can still be used as a first step in evaluating migratory connectivity at broad spatial scales and without knowledge of distant isoscapes. For example, a coarse filter approach would first involve the establishment of isotopic niches across species and populations. Those groups showing good isotopic segregation will be more likely to show separate migratory destinations and so would be ideal candidates for further prioritization using more specific tracking techniques such as the use of geolocators, VHF tags or other intrinsic markers such as genetics or contaminants. As we have demonstrated here using three species of swallows, at the very least, the niche size of a given population should be inversely related to the strength of migratory connectivity with narrow niches indicating a higher probability of connectivity vs broad niches indicating weaker connectivity. We encourage further studies to more formally explore this idea through the isotopic examination of migratory animals or through theoretical modeling.

Our assessment of migratory connectivity for three species of swallow by combining multiple stable isotopes into likelihood assignments provided a useful preliminary assessment of potential regions of non-breeding origin. However, low spatial variation and similarities among broad regions in the Latin American *δ*^2^H and *δ*^13^C feather isoscapes resulted in considerable ambiguity in the assignments to origin. By subsequently applying clustering methods to individual likelihood surfaces, we then defined more spatially discrete regions. Of course, applications using more isotopes and other ancillary information (e.g. morphometrics, trace elements) will likely improve resolution (e.g., [27, 66, 96]) but our results illustrate in general how the stable isotope approach can provide a most parsimonious first means of determining broad patterns of migratory connectivity. Those findings can then be refined by complimentary tracking methods in order to strategically test hypotheses regarding movements over the annual cycle, especially for large (i.e. continental regions). Once clear hypotheses are generated using the isotope techniques we have presented, application of bio-logging devices would further expand our knowledge of swallow connectivity.

Although few in number, geolocators were previously useful in determining migration routes and connectivity for large portions of North American Barn and Tree swallow populations [33, 36] and deployment of these in specific breeding regions will advance our understanding of their migratory connectivity. For example, fitting geolocator tags to Barn Swallows in populations in northern reaches of their range and in more locations in the U.S. (e.g. south, Pacific coast, eastern seaboard) would potentially clarify the connectivity patterns we have postulated for that species and so are encouraged. Further examination of the purported continental migratory divide suggested by Hobson et al. [33] for Barn Swallow with supporting evidence provided in this study for that and the other two swallow species is also required to assess the existence and location of the divide, which we predict runs between Saskatchewan and Ontario to the southwestern US. However, archival tags (e.g. geolocators, GPS) present issues for swallows due to per unit weight and structure, and indeed their use coincides with low recapture rates for some species suggesting other lighter technologies may be more appropriate [33, 35, 97].

The Motus receiver network is a broad-scale (i.e. global) coordinated effort that allows researchers to track movements of animals fit with tags emitting unique VHF signals now over the annual cycle without requiring recapture [98]. While the distribution of receivers in North America is extensive, there are still major spatial gaps in Latin America and so the most effective approach to using this technology to determine spatial connectivity for Nearctic-Neotropical migratory birds including swallows is to tag birds on the non-breeding grounds. Those tagged birds will then have higher chances of detection when they return to their North American breeding grounds where numerous receiving towers exist with more planned. Using isotope techniques, we provide multiple potential regions (i.e. clusters) in Latin America where strategic deployment of Motus tags should occur.

## Supplementary Information

**Additional file 1**. Supporting figures and tables for Methods and Results.

## Data Availability

The datasets used and/or analysed during the current study are available from the corresponding author on reasonable request.
