# Supplementary material for: Multi-isotope (δ2H, δ13C, δ15N) feather profiles and morphometrics inform patterns of migratory connectivity in three species of North American swallows
Source: Mov Ecol. 2023 Aug 1;11:48. doi: 10.1186/s40462-023-00412-2 (PMC10391972; doi:10.1186/s40462-023-00412-2)
Supplement: Supplementary file 1 — Additional file 1. Supporting figures and tables for Methods and Results. [file 40462_2023_412_MOESM1_ESM.docx]

Additional File 1. Fig S1, S2, Table S3.

Supporting figures and tables for Methods and Results

Multi-isotope (*δ*^2^H, *δ*^13^C, *δ*^15^N) feather profiles and morphometrics inform patterns of migratory connectivity in three species of North American swallows

Keith A. Hobson^1,2,3*†^, Kevin J. Kardynal^1,3†^

^1^ Wildlife and Landscape Research Directorate, Environment and Climate Change Canada, Saskatoon, SK, Canada, S7N 3H5

^2^Department of Biology, University of Western Ontario, London, ON, Canada, N6A 5B7

^3^Department of Biology, University of Saskatchewan, Saskatoon, SK, Canada, S7N 5E2


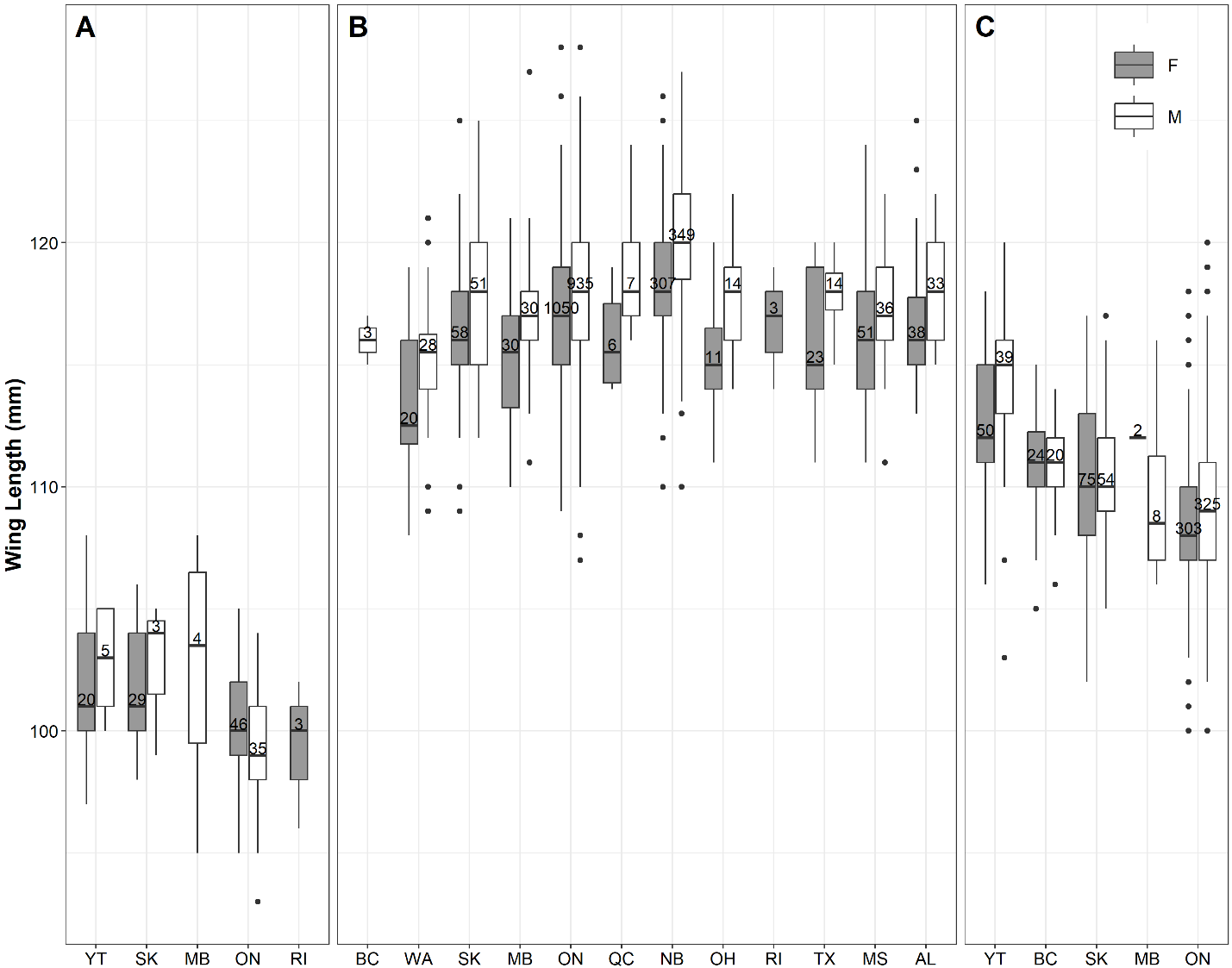


Figure S1. Boxplots showing variation in wing length (mm) across populations and between sexes of adult female (F) and male (M) Bank (A), Barn (B) and Cliff (C) swallows sampled in North America. Midlines within boxes indicate the median isotope value, whiskers extend 1.5x beyond the interquartile range, and points indicate outliers. Values within boxes represent sample sizes. Populations are ordered longitudinally and then latitudinally. Data from some populations were provided by the Bird Banding Laboratory of the Canadian Wildlife Service, Environment and Climate Change Canada. Sampling locations: YT – Yukon, BC – British Columbia, SK – Saskatchewan, MB – Manitoba, ON – Ontario; QC – Quebec, NB – New Brunswick, TX – Texas, MS – Mississippi, AL – Alabama.


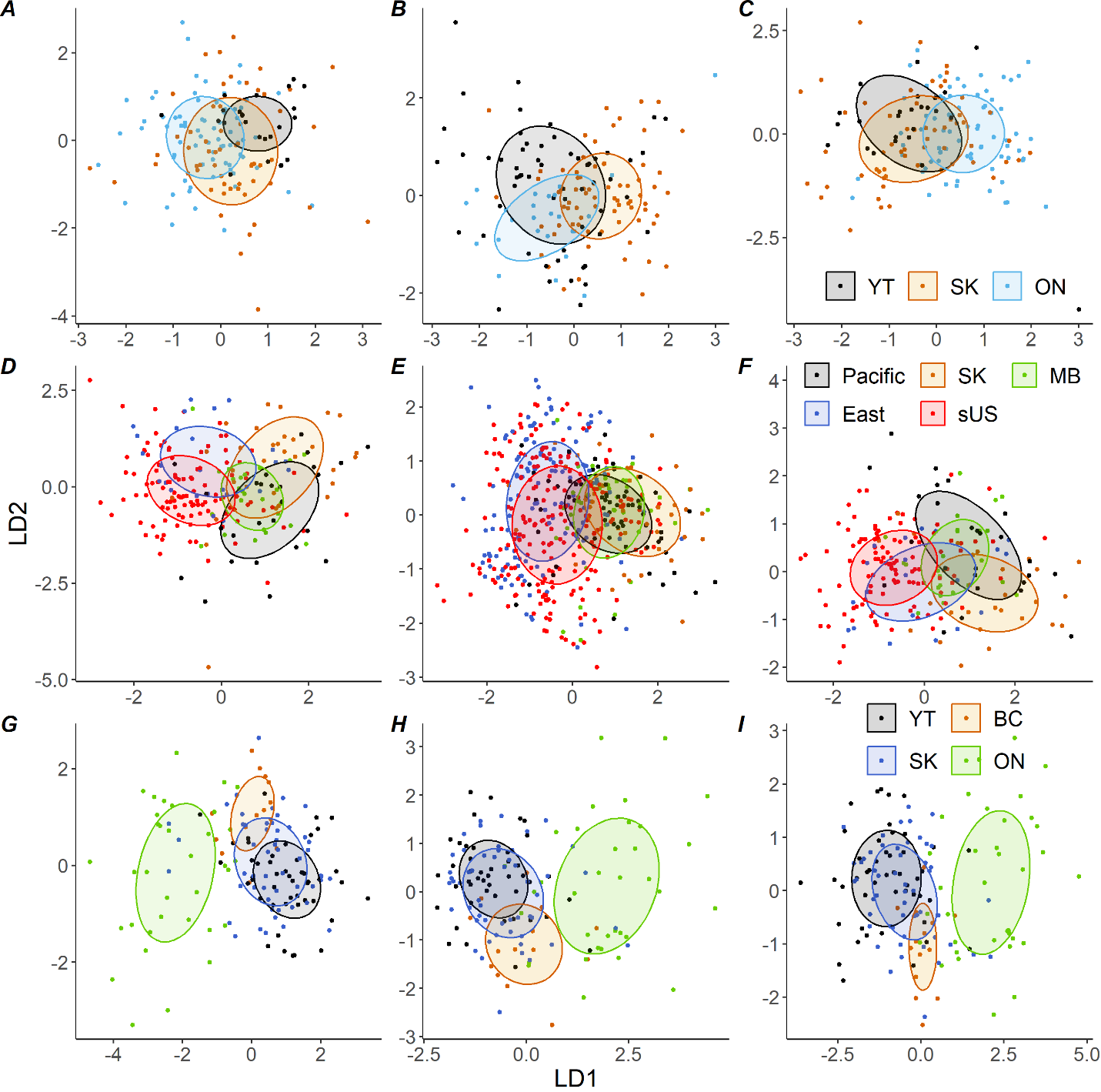


Figure S2. Biplots with 40% Bayesian ellipses showing niche overlap in feather *δ*^13^C–*δ*^2^H–*δ*^15^N–wing length (A, D, G), *δ*^13^C–*δ*^2^H–*δ*^15^N (B, E, H) and *δ*^13^C–*δ*^2^H–wing length (C, F, I) for adult Bank (A–C), Barn (D–F) and Cliff (G–I) swallows sampled in Canada and the United States. Plot axes represent linear discriminant scores from linear discriminant analysis (LDA) conducted on each isotope and wing length combination. Feather isotope values represent non–breeding ground (winter) molt locations. Increasing distance between ellipses indicates greater isotopic and/or wing length dissimilarity. Sampling locations: YT – Yukon, BC – British Columbia, SK – Saskatchewan, ON – Ontario; sampling locations grouped for Barn Swallows: Pacific – BC, Washington, California; SK; MB – Manitoba, East – Ontario, New Brunswick; sUS – southern United States (Colorado, Texas, Mississippi, Alabama). Additional biplots showing isotopic and wing length overlap are presented in Supplementary Material Figure S2.

Table S3. Area and overlap of maximum likelihood fitted 40% Bayesian ellipses estimated using standard ellipse areas assessed on combinations of feather isotope (*δ*^13^C_f_, *δ* ^15^N_f_, *δ* ^2^H_f_) and wing length of multiple populations of Bank, Barn and Cliff swallows sampled in Canada and the United States. Feather isotope values represent non–breeding ground (winter) molt locations. Higher overlap indicates greater isotopic and/or wing length similarity and apparent lower migratory connectivity. Sampling locations: YT – Yukon, BC – British Columbia, SK – Saskatchewan, ON – Ontario; sampling locations grouped for Barn Swallows: Pacific – BC, California, Washington; SK; MB – Manitoba; East – Ontario, New Brunswick; sUS – southern United States (Colorado, Texas, Mississippi, Alabama). Biplots showing isotopic and wing length overlap are presented in Figure 6 and Supplementary Material Figure S2.

| **Species** | **Isotope – Wing Length** | **Populations (Area 1 – Area 2)** | **Area 1** | **Area 2** | **Overlap (%)** |
| --- | --- | --- | --- | --- | --- |
| Bank Swallow (N=162) | *δ*^13^C_f_, *δ* ^15^N_f_, *δ* ^2^H_f_, Wing Length | YT – SK | 2.8 | 4.4 | 39.2 |
|  |  | YT – ON | 2.8 | 2.4 | 17.3 |
|  |  | SK – ON | 2.4 | 4.4 | 21.0 |
|  | *δ*^13^C_f_, *δ*^15^N_f_, *δ*^2^H_f_ | YT – SK | 3.0 | 3.9 | 37.5 |
|  |  | YT – ON | 3.0 | 2.4 | 32.2 |
|  |  | SK – ON | 2.4 | 3.9 | 39.4 |
|  | *δ*^13^C_f_, *δ*^2^H_f_, Wing Length | YT – SK | 3.8 | 3.8 | 60.1 |
|  |  | YT – ON | 2.5 | 3.8 | 17.7 |
|  |  | SK – ON | 3.8 | 2.5 | 22.4 |
| Barn Swallow (N=222) | *δ*^13^C_f_, *δ* ^15^N_f_, *δ* ^2^H_f_, Wing Length | Pacific – SK | 4.5 | 4.6 | 28.4 |
|  |  | Pacific – MB | 4.5 | 1.8 | 25.8 |
|  |  | Pacific – East | 4.5 | 2.9 | 0.5 |
|  |  | Pacific – sUS | 4.5 | 2.6 | 0.9 |
|  |  | SK – MB | 4.6 | 1.8 | 25.5 |
|  |  | SK – East | 4.6 | 2.9 | 8.3 |
|  |  | SK – sUS | 4.6 | 2.6 | 1.1 |
|  |  | MB – East | 1.8 | 2.9 | 11.6 |
|  |  | MB – sUS | 1.8 | 2.6 | 5.3 |
|  |  | East – sUS | 2.9 | 2.6 | 28.8 |
|  | *δ*^13^C_f_, *δ*^15^N_f_, *δ*^2^H_f_ | Pacific – SK | 2.5 | 3.2 | 45.6 |
|  |  | Pacific – MB | 2.5 | 2.1 | 61.1 |
|  |  | Pacific – East | 2.5 | 3.4 | 14.8 |
|  |  | Pacific – sUS | 2.5 | 3.2 | 8.2 |
|  |  | SK – MB | 3.2 | 2.1 | 40.2 |
|  |  | SK – East | 3.2 | 3.4 | 3.5 |
|  |  | SK – sUS | 3.2 | 3.2 | 0.6 |
|  |  | MB – East | 2.1 | 3.4 | 13.1 |
|  |  | MB – sUS | 2.1 | 3.2 | 4.5 |
|  |  | East – sUS | 3.4 | 3.2 | 54.5 |
|  | *δ*^13^C_f_, *δ*^2^H_f_, Wing Length | Pacific – SK | 3.6 | 5.5 | 31.5 |
|  |  | Pacific – MB | 3.6 | 2.2 | 27.6 |
|  |  | Pacific – East | 3.6 | 2.7 | 6.5 |
|  |  | Pacific – sUS | 3.6 | 2.4 | 0.7 |
|  |  | SK – MB | 5.5 | 2.2 | 26.4 |
|  |  | SK – East | 5.5 | 2.7 | 10.4 |
|  |  | SK – sUS | 5.5 | 2.4 | 0.8 |
|  |  | MB – East | 2.2 | 2.7 | 30.5 |
|  |  | MB – sUS | 2.2 | 2.4 | 7.6 |
|  |  | East – sUS | 2.7 | 2.4 | 44.6 |
| Cliff Swallow (N=149) | *δ*^13^C_f_, *δ* ^15^N_f_, *δ* ^2^H_f_, Wing Length | YT – BC | 2.4 | 1.5 | 0.0 |
|  |  | YT – SK | 2.4 | 3.0 | 58.0 |
|  |  | YT – ON | 2.4 | 5.0 | 0.0 |
|  |  | BC – SK | 1.5 | 3.0 | 11.6 |
|  |  | BC – ON | 1.5 | 5.0 | 0.0 |
|  |  | SK – ON | 3.0 | 5.0 | 0.0 |
|  | *δ*^13^C_f_, *δ*^15^N_f_, *δ*^2^H_f_ | YT – BC | 2.1 | 5.1 | 2.9 |
|  |  | YT – SK | 2.1 | 2.8 | 58.0 |
|  |  | YT – ON | 2.1 | 5.6 | 0.0 |
|  |  | SK – BC | 2.8 | 5.1 | 15.2 |
|  |  | BC – ON | 5.1 | 5.6 | 0.0 |
|  |  | SK – ON | 2.8 | 5.6 | 0.0 |
|  | *δ*^13^C_f_, *δ*^2^H_f_, Wing Length | YT – BC | 3.0 | 1.1 | 0.0 |
|  |  | YT – SK | 3.0 | 2.7 | 45.5 |
|  |  | YT – ON | 3.0 | 4.9 | 0.0 |
|  |  | BC – SK | 1.1 | 2.7 | 0.0 |
|  |  | BC – ON | 1.1 | 4.9 | 0.0 |
|  |  | SK – ON | 2.7 | 4.9 | 0.0 |
